# Role of C/EBPβ Transcription Factor in Adult Hippocampal Neurogenesis

**DOI:** 10.1371/journal.pone.0024842

**Published:** 2011-10-07

**Authors:** Marta Cortes-Canteli, Diana Aguilar-Morante, Marina Sanz-SanCristobal, Diego Megias, Angel Santos, Ana Perez-Castillo

**Affiliations:** 1 Instituto de Investigaciones Biomedicas “Alberto Sols”, Consejo Superior de Investigaciones Cientificas-Universidad Autonoma de Madrid, Madrid, Spain; 2 Centro de Investigación Biomédica en Red sobre Enfermedades Neurodegenerativas (CIBERNED), Madrid, Spain; 3 Centro Nacional de Investigaciones Oncologicas, Madrid, Spain; 4 Departamento de Bioquimica y Biologia Molecular, Facultad de Medicina, Universidad Complutense de Madrid, Madrid, Spain; University of South Florida, United States of America

## Abstract

**Background:**

The dentate gyrus of the hippocampus is one of the regions in which neurogenesis takes place in the adult brain. We have previously demonstrated that CCAAT/enhancer binding protein β (C/EBPβ) is expressed in the granular layer of the dentate gyrus of the adult mouse hippocampus. Taking into account the important role of C/EBPβ in the consolidation of long term memory, the fact that newborn neurons in the hippocampus contribute to learning and memory processes, and the role of this transcription factor, previously demonstrated by our group, in regulating neuronal differentiation, we speculated that this transcription factor could regulate stem/progenitor cells in this region of the brain.

**Methodology/Principal Findings:**

Here, we show, using *C/EBPβ* knockout mice, that C/EBPβ expression is observed in the subset of newborn cells that proliferate in the hippocampus of the adult brain. Mice lacking *C/EBPβ* present reduced survival of newborn cells in the hippocampus, a decrease in the number of these cells that differentiate into neurons and a diminished number of cells that are proliferating in the subgranular zone of the dentate gyrus. These results were further confirmed *in vitro*. Neurosphere cultures from adult mice deficient in *C/EBPβ* present less proliferation and neuronal differentiation than neurospheres derived from wild type mice.

**Conclusions/Significance:**

In summary, using *in vivo* and *in vitro* strategies, we have identified C/EBPβ as a key player in the proliferation and survival of the new neurons produced in the adult mouse hippocampus. Our results support a novel role of C/EBPβ in the processes of adult hippocampal neurogenesis, providing new insights into the mechanisms that control neurogenesis in this region of the brain.

## Introduction

CCAAT/enhancer binding protein β (C/EBPβ) is a member of the transcriptional factor family consisting of six functionally and structurally related basic leucine zipper DNA-binding proteins [1]. C/EBPβ is expressed in numerous tissues, including the liver, adipose tissue, ovary, lung, kidney, mammary gland, and hematopoietic tissues (reviewed in [2]) and regulates many genes involved in different cell processes including metabolism, hematopoiesis, adipogenesis, the immune response, and morphogenesis [2], [3]. It has been also shown that C/EBPβ mRNA is expressed in the central nervous system of adult mice [4], [5] and several studies, including those from our laboratory, have suggested that this protein may have important functions in the brain. Our work has revealed a previously unknown role for C/EBPβ in the nervous system. We have demonstrated that C/EBPβ regulates the expression of several genes involved in inflammatory processes and brain injury [6], and mice lacking *C/EBPβ* showed a reduced inflammatory response after kainic acid injection and exhibited a dramatic reduction in pyramidal cell loss in the CA1 and CA3 subfields of the hippocampus [7]. It has been also shown that C/EBPβ plays an important role in the consolidation of long-term memory, suggesting a very important role for this protein in the hippocampus [8], [9] and Menard et al have defined a MEK-C/EBP pathway as being essential for the differentiation of cortical progenitor cells into postmitotic neurons [10]. In this regard, we have demonstrated that C/EBPβ serves as a critical factor in neuronal differentiation [11].

In the central nervous system, developing neurons are derived from quiescent multipotent or neural stem cells [12]. The hippocampus is a unique structure in that it is one of the two brain regions where adult neurogenesis persists throughout adulthood. New neurons are continuously generated in the subgranular zone (SGZ) of the dentate gyrus (DG) of the hippocampus, migrate into the granule layer, and differentiate into new dentate granule cell neurons [12], [13]. It has recently been demonstrated that these new generated neurons subsequently integrate into memory networks [14]. Considering the important role of C/EBPβ in the consolidation of long term memory and its role in regulating neuronal differentiation, we speculated that this transcription factor could have a role in hippocampal stem/progenitor cells. Here, we demonstrate that C/EBPβ is expressed in the DG of the hippocampus and has a key role in regulating the proliferation and differentiation of these cells *in vitro* and *in vivo*.

## Results

### C/EBPβ is expressed during postnatal development in granular neurons of the DG of the hippocampus

**Figure 1:**
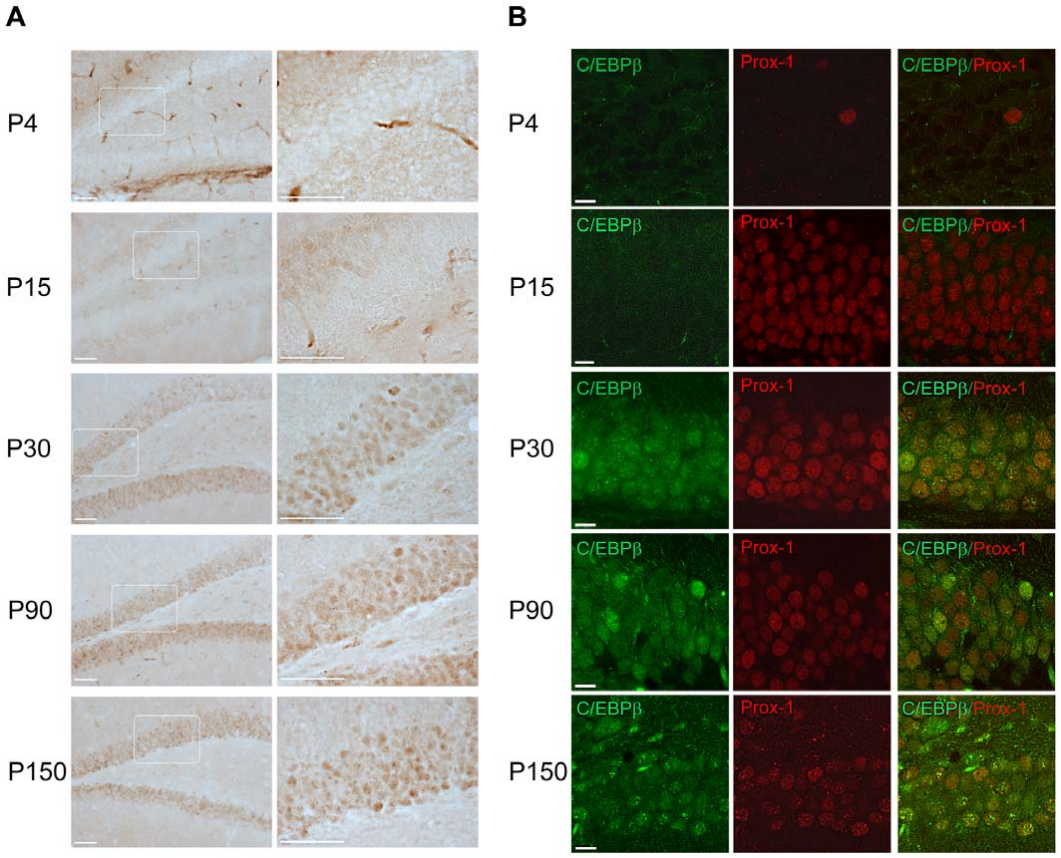
C/EBPβ is expressed in the DG of the hippocampus at different postnatal stages. (A) C/EBPβ immunohistochemical analysis of coronal brain sections prepared form wild type mice of four days (P4), 15 days (P15), 30 days (P30), three months (P90) and five months (P150) of age show that C/EBPβ protein begins to increase at P15 in the DG, reaching the maximum expression at P30. No C/EBPβ staining is detected at P4. Right panels are higher magnifications of the granule layer (white boxes in the left panels). Scale bars, 50 µm. (B) Double immunofluorescent labeling and confocal microscopic analysis show colocalization of C/EBPβ (green) with the granule cell marker Prox-1 (red). Scale bars, 10 µm.

C/EBPβ is implicated in the differentiation of adipocytes and macrophages, among other cell types [2], and we have previously demonstrated that this transcription factor induces neuronal differentiation [11]. Also, we found that its expression is strongly increased in the neurons of the granule layer of the adult hippocampus after brain injury [7]. These data, together with the fact that this transcription factor plays an important role in hippocampal function [15], prompted us to investigate whether C/EBPβ had a role in hippocampal neurogenesis. To this end, we first analyzed the expression and localization of this protein in the hippocampus during postnatal development and in adult mice. We determined C/EBPβ expression in the DG of wild type mice at postnatal day four, day 15, day 30, day 90 and day 150 (Fig. 1). We found no C/EBPβ expression at four days of age in this specific area of the brain (the staining surrounding the DG seen in the P4 panels of Fig. 1A is non-specific). However, the protein levels of this transcription factor began to slightly increase at postnatal day 15, reached a peak at 30 days of age and were maintained high in adult mice (Fig. 1A). C/EBPβ expression in the hippocampus was mainly localized in the nuclei of granular neurons of the DG as determined by its colocalization with the homeobox prospero-like protein (Prox-1), a specific granule cell marker (Fig. 1B).

### C/EBPβ is expressed in the neural stem cell population in the adult hippocampus

**Figure 2:**
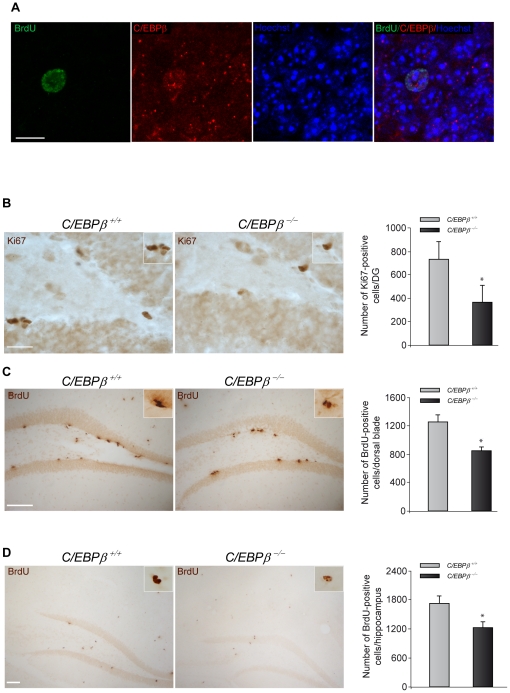
*C/EBPβ^−/−^* mice present less proliferation and survival of newborn cells in the hippocampus. (A) C/EBPβ is expressed in neural stem cells of the dentate gyrus. Representative images of double-immunohistochemistry showing BrdU-positive cells (green) expressing C/EBPβ (red) in the granule layer of the hippocampus of wild type mice 28 days after BrdU injection. Nuclei were counterstained with Hoechst (blue). Scale bar, 10 µm. (B) Representative images showing Ki67 immunohistochemistry analysis of the DG of postnatal day 30 *C/EBPβ^+/+^* and *C/EBPβ^−/−^* mice. Quantification of the Ki67-positive cells in the SGZ revealed that *C/EBPβ^−/−^* mice presented a significant reduction in cells positive for this proliferative marker. Scale bar, 25 µm. BrdU was administered to three month-old C/EBP*β* ^+/+^ and C/EBP*β* ^−/−^ mice as explained in Materials and Methods. The animals were sacrificed one (C) or 28 days (D) after the last BrdU injection to measure proliferation or survival, respectively. BrdU immunohistochemistry and quantification analysis revealed significantly less BrdU-positive cells in the dorsal part of the dentate gyrus in *C/EBPβ^−/−^* compared to *C/EBPβ^+/+^* mice (C). Also, the number of surviving BrdU-positive cells in the entire hippocampus was significantly reduced in *C/EBPβ^−/−^* mice (D). Scale bars, 100 µm. Insets show higher magnification. Values represent the mean ± S.E from four different animals. *P≤0.05.

We then examined whether C/EBPβ is expressed in the newborn cells. To this end, adult wild type mice were injected with the DNA analog BrdU to identify any cell that was dividing, and 28 days after the injection we analyzed whether any BrdU-positive cell present in the hippocampus also contained C/EBPβ. Double-immunofluorescence analysis showed that cells that have incorporated BrdU were also expressing C/EBPβ (Fig. 2A), demonstrating that newly born cells express this transcription factor.

### Decreased proliferation and survival of neural stem cells in the adult hippocampus of *C/EBPβ ^−/−^* mice

C/EBPβ promotes proliferation of several cell types, such as hepatocytes [16] and epithelial cells [17]. Thus, we next investigated whether the loss of C/EBPβ affected cell proliferation in the hippocampal DG. We identified the proliferating cells with the cell cycle protein Ki67, which has been defined as an accurate endogenous proliferation marker of SGZ precursors [18]. We found that 30-day old *C/EBPβ ^−/−^* mice presented a 50% decrease in the number of cells positive for this nuclear antigen in the SGZ when compared to *C/EBPβ ^+/+^* control littermates (Fig. 2B). This significant decrease demonstrates that C/EBPβ has an important effect on the proliferation of progenitor cells in the adult SGZ. These results were further confirmed by determining the number of BrdU-positive cells in the SGZ of the dentate gyrus of three month-old *C/EBPβ ^+/+^* and *C/EBPβ ^−/−^* mice, one day after the last injection of BrdU. We counted both, the dorsal and ventral blades of the DG. As shown in Figure 2C, we found a reduction in the number of BrdU-positive cells in knockout mice, which was only apparent in the dorsal blade of the SGZ of the DG. These regional differences in the proliferation of progenitor cells in the dentate gyrus of adult mice have been previously shown by other authors and in different paradigms [19], [20], [21].

We next analyzed whether this protein might influence the survival rate of the newborn cells in the adult hippocampus. Three month-old *C/EBPβ ^+/+^* and *C/EBPβ ^−/−^* adult mice were injected with BrdU and the total number of BrdU-positive cells in the whole hippocampus was quantified 28 days after the last injection. We chose this time point because the migration and differentiation of the newborn cells is finished and the number of BrdU-positive cells becomes stable four weeks after the last BrdU injection [21]. Our results show that the number of surviving neural stem cells in the entire hippocampus was significantly lower in the mice lacking *C/EBPβ* (Fig. 2D), indicating that this protein also plays a role in the survival rate of newborn adult neural stem cells.

The difference between *C/EBPβ ^+/+^* and *C/EBPβ ^−/−^* mice 24 hours after BrdU administration was only found in the dorsal blade (Fig. 2C) and not in the whole hippocampus as happened at 28 days (Fig. 2D). This fact may represent a rapid effect of C/EBPβ on proliferation in this particular area of the dentate gyrus of the hippocampus as well as a long effect on survival in the whole hippocampus.

### C/EBPβ enhances the number of newborn cells expressing Prox-1

**Figure 3:**
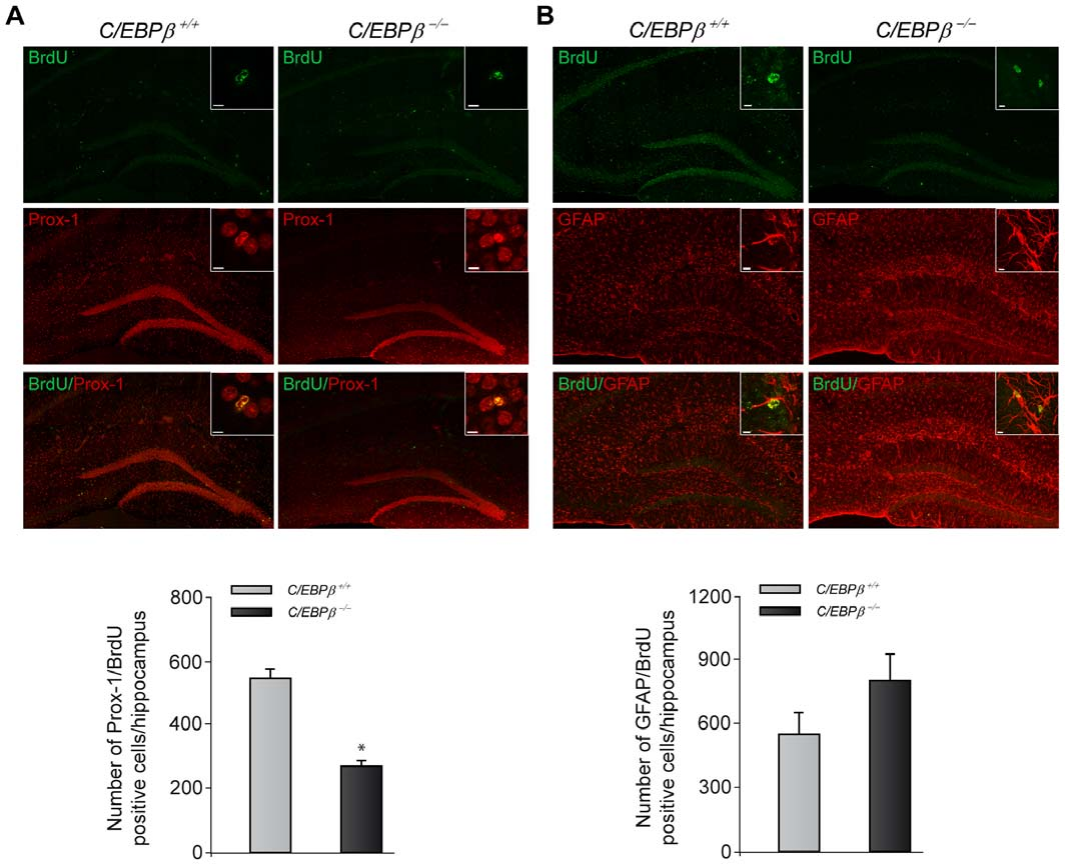
Phenotypic analysis of BrdU-positive cells revealed less surviving neurons in the hippocampus of *C/EBPβ^−/−^* compared to *C/EBPβ^+/+^* mice. Coronal sections from *C/EBPβ^+/+^* and *C/EBPβ^−/−^* mice were double labeled with BrdU (green) and neuronal or astrocytic markers (red). Subsequent biomapping experiments by confocal microscopy were carried out as described in Materials and Methods. Quantification analysis of the whole hippocampus revealed that 28 days after BrdU administration the percentage of BrdU positive cells that express the granule cell marker Prox-1 (A) is significantly decreased in the hippocampus of *C/EBPβ^−/−^* mice. In contrast, the percentage of cells that coexpress BrdU and the astrocytic marker GFAP is increased in the hippocampus of *C/EBPβ^−/−^* mice (B). Insets represent examples of double-positive cells at high power magnification. Scale bars, 5 µm. Values represent the mean ± S.E from two-four different biomappings/group. *P≤0.05.

We have previously shown that C/EBPβ induces neuronal differentiation [11]. Also, its phosporylation has been shown to be essential for cortical precursor cultures to become neurons [10] and not astrocytes. Therefore, we analyzed if the observed decrease in surviving newborn cells found in *C/EBPβ ^−/−^* mice (Fig. 2D) corresponds to a decrease generation of neurons or astrocytes. For that purpose, we performed double-immunofluorescence analysis to detect cells containing both BrdU and specific neuronal/astrocytic markers, followed by biomapping analysis. This approach allowed the quantification of all the double-positive cells in the entire hippocampus. Interestingly, the total number of BrdU-positive cells that become postmitotic granular neurons (identified with the neuronal marker Prox-1), was significantly reduced by 50% in *C/EBPβ ^−/−^* mice (Fig. 3A). On the contrary, the number of astrocytes produced was increased in mice lacking *C/EBPβ*, although it did not reach significance (*P* = 0.2. Fig. 3B). These results show that C/EBPβ promotes neurogenesis over gliogenesis in neural progenitors of the adult hippocampus.

### Neurospheres derived from adult *C/EBPβ ^−/−^* mice show reduced proliferation and neuronal differentiation

**Figure 4:**
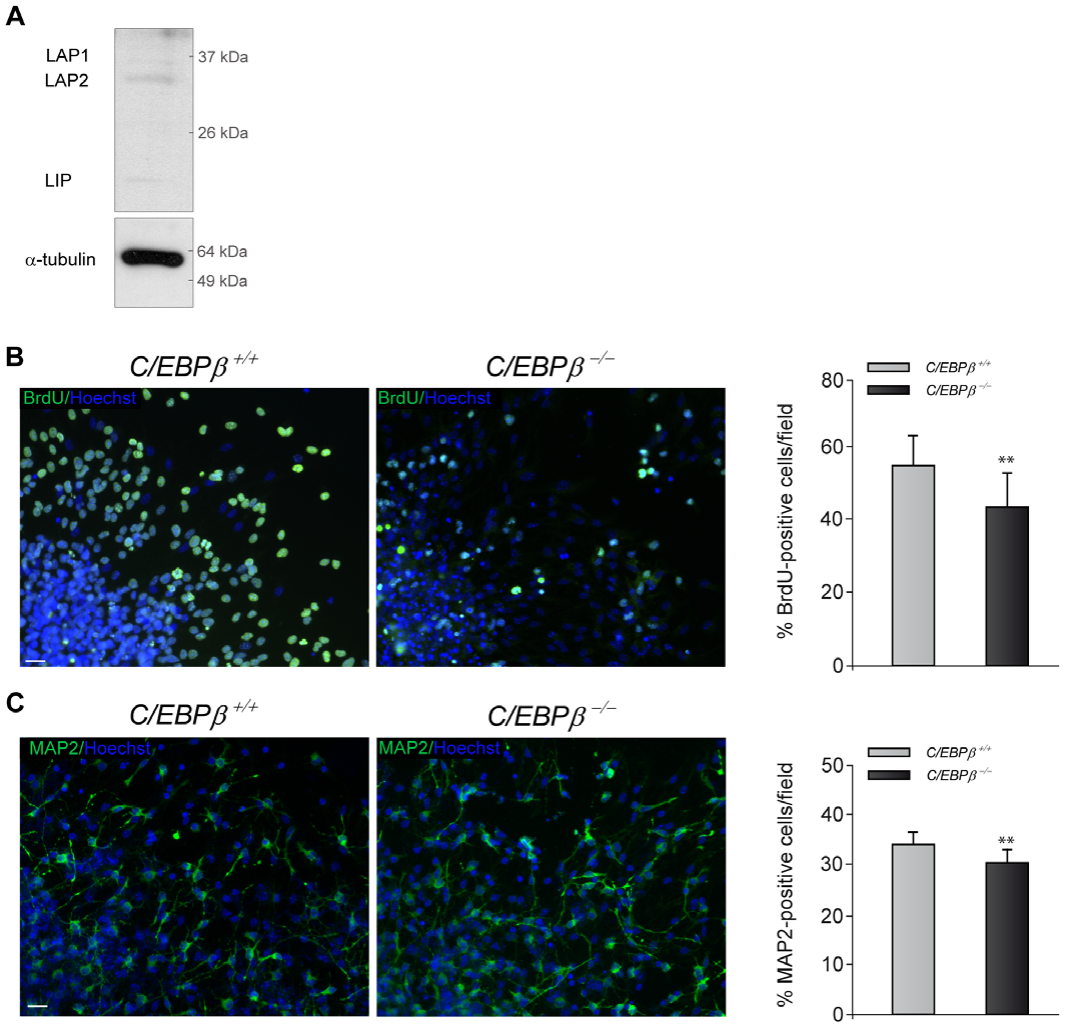
Reduced proliferation and neuronal differentiation in neurospheres isolated from adult *C/EBPβ^−/−^* compared to *C/EBPβ^+/+^* mice. (A) Representative Western blot showing the presence of the different C/EBPβ isoforms (LAP1, LAP2 and LIP) in NS cultures established from adult mice. (B) Immunocytochemistry analysis showing the BrdU incorporation in *C/EBPβ^−/−^* and *C/EBPβ^+/+^* NS cultures plated for 24 h in proliferation medium. Quantification analysis show a significantly reduction in the number of BrdU-positive cells in *C/EBPβ^−/−^* cultures compared to wild type NS cultures. (C) *C/EBPβ^−/−^* and *C/EBPβ^+/+^* NS cultures were plated for 72 h in differentiation medium and MAP2 immunocytochemistry was performed as described in Material and Methods. Quantification analysis shows that the percentage of MAP2 positive cells in *C/EBPβ^−/−^* NS cultures was significantly reduced. Nuclei were counterstained with Hoechst (blue). Scale bars, 25 µm. Values represent the mean ± S.E. **P≤0.01.

One of the characteristics of neural stem cells is their ability of growing *in vitro* as spheres [22]. To confirm the *in vivo* effects on neuronal differentiation (Fig. 3) and proliferation (Fig. 2) we found on adult hippocampal neurogenesis in *C/EBPβ ^−/−^* mice, we next performed *in vitro* studies with neurosphere (NS) cultures. We established NS cultures from adult *C/EBPβ^+/+^* and *C/EBPβ^−/−^* mice and compared their proliferation and differentiation properties. First, we determined whether neural stem cells conserved C/EBPβ expression (Fig. 2A) when isolated and cultured *in vitro*. Western blot analysis of NS established from wild type mice confirmed that these cells indeed express the three isoforms of C/EBPβ: liver activating protein 1 (LAP1), LAP2 and liver inhibitory protein (LIP), being LAP2 the most prominent (Fig. 4A). Then, we analyzed whether, as happened *in vivo* (Fig. 2), the proliferation rate was also affected by the lack of C/EBPβ *in vitro*. NS isolated from *C/EBPβ ^−/−^* mice presented a significant decrease in BrdU incorporation (Fig. 4B) confirming that C/EBPβ is acting as a proliferative stimulus in adult neural stem cells. To further confirm that the presence of C/EBPβ is a determinant factor on progenitor cells proliferation, we repeated the same proliferation assay but on NS isolated at postnatal day 2, when C/EBPβ is absent (Fig. 1). We found no differences in the total number of BrdU-stained cells between *C/EBPβ^+/+^* and *C/EBPβ^−/−^* NS (data not shown). This result further reinforces the idea that C/EBPβ is required to maintain the hippocampal/progenitor cell population.

Lastly, we analyzed the number of neurons in cultures derived from wild type and *C/EBPβ* deficient animals. For that purpose, NS cultures established from adult *C/EBPβ^+/+^* and *C/EBPβ ^−/−^* mice were plated in the absence of growth factors, and the percentage of MAP2-positive cells was analyzed. In accordance with the result obtained *in vivo* with the biomapping experiment (Fig. 3A), the total number of mature neurons, identified by MAP2 staining, was significantly lower in NS isolated from *C/EBPβ ^−/−^* mice compared to wild type mice (Fig. 4C). To confirm that the reduction in the number of MAP2-positive cells is not due to a general decreased in the proliferation rate in *C/EBPβ ^−/−^* NS, we also analyzed the percentage of GFAP and 2′, 3′-cyclic nucleotide 3′-phospho- diesterase (CNPase) cells, to identify astrocytes and oligodendrocytes, respectively. We did not detect any significant difference in any of these two markers in NS isolated from wild type and knockout animals (GFAP-positive cells/field: 27.24±6.31% in WT *vs* 25.59±8.12% in KO; CNPase-positive cells/field: 26.66±6.49% in WT *vs* 18.79±4.60% in KO). These results indicate that C/EBPβ is specifically inducing differentiation toward a neuronal phenotype. In agreement with our results, Menard et al have shown that the MEK-C/EBPβ pathway enhances neurogenesis while inhibiting gliogenesis [10].

These *in vitro* studies confirm the *in vivo* results and suggest an important role for C/EBPβ in adult neurogenesis.

## Discussion

In the adult, neurogenesis in the hippocampus is restricted to the SGZ of the DG [23]. Although the role of C/EBPβ in different brain functions has been investigated during the last years, there is no information regarding the effect of this transcription factor on neurogenesis in the adult brain. The results of the present study show that C/EBPβ plays a role in the adult hippocampal neurogenesis. C/EBPβ is expressed in the newly generated neural stem cells of the DG, and loss of C/EBPβ results in reduced cell proliferation and impaired differentiation and survival of newly generated neurons in the adult hippocampal DG *in vivo*. C/EBPβ effects on neurogenesis are also evident *in vitro* by a decrease of both proliferation and differentiation toward a neuronal phenotype in neurospheres derived from *C/EBPβ ^−/−^* mice.

Our observation that C/EBPβ is highly expressed in the DG of the adult hippocampus is consistent with previous findings showing that C/EBPβ mRNA is expressed primarily in neurons throughout the mature brain, with high levels in the hippocampus [5]. Also, C/EBPβ protein has been detected in hippocampal neurons [24] and previous studies from our group showed that the levels of this transcription factor increase in granular neurons of the DG after brain injury [7].

Neurogenesis can be regulated at multiple points, including proliferation, differentiation, and migration, processes that are controlled by a number of intracellular and extracellular signaling cues [25], [26]. A correct balance between these processes is key to control the scarce replacement of neurons that takes place in the adult brain. Therefore, the identification of new factors implicated in these processes will help to understand the molecular basis of the adult neurogenesis and how it could be controlled. We here show that C/EBPβ regulates two of these processes, proliferation and neuronal differentiation.

It has been widely shown that C/EBPβ plays a critical role in regulating proliferation as well as differentiation of many cell types. C/EBPβ promotes proliferation of hepatocytes [16], epithelial cells [17], and participates in the proliferation of Schwann cells [27]. However, in other systems this transcription factor seems to play an anti-proliferative role [28], [29]. The data from the present experiments indicate that adult neurogenesis was decreased in the SGZ of *C/EBPβ ^−/−^* mice, since BrdU incorporation was significantly diminished in the hippocampus and there was a decrease in the Ki67 staining in the SGZ of *C/EBPβ ^−/−^* mice. Hence, our studies demonstrate that C/EBPβ induces proliferation in the neurogenic niche of the adult hippocampus, then identifying a new function for this protein.

As commented above, C/EBPβ is also an important regulator of differentiation in different cell types, including neurons. Sterneck and Johnson demonstrated that C/EBPβ is activated when PC12 cells undergo neuronal differentiation [5]. Menard et al have defined a MEK-C/EBP pathway as being essential for the differentiation of cortical progenitor cells into postmitotic neurons [10]. We have also demonstrated that overexpression of C/EBPβ in a neuroblastoma cell line induces neuronal differentiation [11]. Here we show for the first time that this transcription factor is also critical for the neural stem cells to become neurons in the hippocampus of the rodent adult brain.

The role of neurogenesis in the hippocampus is not completely clear, but it has been suggested to be involved in memory formation [30], [31]. Poor performance in hippocampal-dependent memory tasks has been found in mice where adult neurogenesis had been depleted, although there is some discrepancy in this field [32]. Also, an age-related decrease in neurogenesis has been postulated to be linked to age-related memory deficits [33]. In this regard, it has been shown that C/EBPβ plays an important role in the consolidation of long-term memory, suggesting a very important role for this protein in the hippocampus. Not only C/EBPβ expression is enhanced during long term potentiation [8], [9] but it also co-localizes in hippocampal neurons with the phosphorylated cAMP response element-binding protein (CREB) [15], an essential transcription factor in memory consolidation. In addition, it has been suggested that CREB activates C/EBP expression [24], suggesting a CREB-C/EBP pathway in the memory formation pathway, and therefore, an important role for C/EBPβ in this process.

Neural stem cells can differentiate into neurons, astrocytes and oligodendrocytes [12], [34]. Here, we show for the first time that C/EBPβ is involved in the control of neural stem cell differentiation. Our results support a novel role of C/EBPβ in the processes of adult hippocampal neurogenesis, providing new insights into the mechanisms of neurogenesis regulation and suggesting that its use may have important implications for directing controlled differentiation of neural stem cells toward a neuronal lineage.

## Materials and Methods

### Animals

*C/EBPβ ^+/+^* and *C/EBPβ ^−/−^* mice were generated from heterozygous breeding pairs, kindly provided by Dr. C.M. Croniger and Dr. R.W. Hanson (Case Western Reserve University, Cleveland, Ohio) [35]. Genotypes were identified based on genomic PCR with DNA prepared from tail using the REDExtract-N-AmpTM Tissue PCR kit (XNAT kit, Sigma, St. Louis, MO). All procedures with animals were specifically approved by ‘Ethics Committee for Animal Experimentation’ of the Instituto de Investigaciones Biomedicas (CSIC-UAM), license number SAF2010/16365, and carried out in accordance with the protocols issued which followed National (normative 1201/2005) and International recommendations (normative 86/609 from the European Communities Council). Adequate measures were taken to minimize pain or discomfort of animals.

### BrdU administration and quantification

Adult male *C/EBPβ ^+/+^* and *C/EBPβ ^−/−^* mice (∼ three month-old) received during three consecutive days two intraperitoneal daily injections (six hours apart) of the DNA base analog 5-Bromo-2′-deoxyuridine (BrdU; Sigma; 50 mg/kg body weight). Animals were killed one and 28 days after the last BrdU injection, to measure proliferation and survival of the newborn cells, respectively. The total number of BrdU-positive cells was determined in every 8th 30 µm coronal sections from four animals/group, covering the unilateral hippocampus from Bregma −1.58 mm to −2.54 mm, using a 63x objective to avoid oversampling errors. Cells touching sample edges were not counted. BrdU-positive cells in the SGZ (proliferation group) or throughout the whole hippocampus (survival group) were counted. The resulting number was multiplied by 8 to have an approximate total number of BrdU-positive cells in the entire hippocampus.

### Tissue preparation and immunohistochemistry

Animals (n = 2–4 mice/group) were anaesthetized and perfused transcardially with 4% paraformaldehyde solution. The brains were removed, postfixed in the same solution at 4°C overnight, cryoprotected in the paraformaldehyde solution containing 30% sucrose, frozen, and 30 µm coronal sections were obtained in a cryostat. Immunohistochemistry using the diaminobenzidine method and double-immunofluorescence were performed as previously described [7] and the slides were examined with a Zeiss Axiophot microscope equipped with an Olympus DP-70 digital camera or a Leica Confocal SP5 microscope, respectively. For the BrdU immunohistochemistry, the sections were pre-treated with 55% formamide/2x SSC for one hour at 55°C, followed by 30 min of 2N HCl at 37°C and finally washed in 0.1 M borate buffer for 20 min.

The antibodies used were: rabbit polyclonal anti-Prox-1 (Chemicon, Temecula, CA), anti-glial fibrillary acidic protein (GFAP; Dako, Glostrup, Denmark) and anti-Ki67 (Novocastra Laboratories, UK); mouse monoclonal anti-C/EBPβ (clone A16, Abcam, Cambridge, UK) and rat monoclonal anti-BrdU (Abcam). For double-immunofluorescence, anti-rat-Alexa Fluor 488 and anti-rabbit-Alexa Fluor 647 (Invitrogen, Carlsbad, CA) were used. Mouse monoclonal immunohistochemistry was performed using the Mouse on Mouse immunodetection kit (Vector Labs, Burlingame, CA) following Manufacturer's instructions and as previously described [7]. Hoechst (Invitrogen) was used to counterstain nuclei.

The total number of Ki67-positive cells in the SGZ was determined as detailed above for the BrdU quantification. Cells present in the two-nucleus-wide band below and above the SGZ were included in the quantification.

Biomappings from the double immunofluorescence of BrdU/neural markers were obtained with a Leica Confocal TCS-SP5 (AOBS) microscope by using the Matrix Screening application. Z-stack tile-scans covering the entire hippocampus were acquired with a 63x 1.4 N.A.HCX PL APO immersion objective. After image reconstruction, the total number of BrdU/Prox-1 or BrdU/GFAP positive cells in the entire hippocampus was carefully quantified by hand, graphed and compared between *C/EBPβ ^+/+^* and *C/EBPβ ^−/−^* mice.

### Neurosphere cultures

Neurosphere (NS) cultures were derived from adult mice (∼ four month-old). Briefly, 4 mice per culture were decapitated, brains removed and the hippocampus and subventricular zone were dissected, minced and dissociated with DMEM (Invitrogen) containing glutamine, gentamicin and fungizone. After treatment with trypsin-EDTA, hialuronidase and DNAse, myelin was removed by using DPBS (Invitrogen). Cells were seeded into six-well dishes and cultured in DMEM:F12 (1∶1, Invitrogen) containing 20 ng/ml epidermal growth factor (EGF, Peprotech, London, UK), 20 ng/ml fibroblast growth factor (FGF, Peprotech) and B27 medium (Invitrogen). After ∼10 days in culture, some NS were centrifuged five min at 1000× g, washed with phosphate-buffered-saline and protein was extracted in RIPA buffer. Twenty µg of total protein was loaded on a 10% SDS-PAGE gel and western blot analysis to detect C/EBPβ was performed as previously described [7]. Mouse monoclonal antibody against α-tubulin (Sigma) was used as loading control. Some NS cultures were treated with 10 µM BrdU (Sigma) for 24 h and plated onto 100 µg/ml poly-L-lysine (Sigma) coated coverslips for another 24 h in normal proliferative conditions to assess proliferation. In order to determine the ability of *C/EBPβ^+/+^* and *C/EBPβ^−/−^* to produce neurons, NS from 10-day old cultures were plated for 72 h onto 100 µg/ml poly-L-lysine coated coverslips in the absence of exogenous growth factors. Then, cells were processed for immunocytochemistry for microtubule-associated protein 2 (MAP2) to identify the neurons.

### Immunocytochemistry

Cells were processed for immunocytochemistry as previously described [36]. Briefly, at the end of the treatment period, NS cultures, grown on glass cover-slips in 24-well cell culture plates, were washed with phosphate-buffered-saline and fixed for 30 min with 4% paraformaldehyde. Then the cells were permeabilized and blocked with 0.5% Triton X-100 and 5% goat serum for 30 min at 37°C. In the case of the BrdU immunocytochemistry, cells were pretreated five min with 2N HCl. After one hour incubation with the corresponding primary antibody (mouse monoclonal anti-MAP2 (Sigma) or rat-monoclonal anti-BrdU (Abcam)), cells were washed with phosphate-buffered-saline and incubated with the correspondent Alexa-488 secondary antibody and Hoechst (Invitrogen) for 45 min at 37°C. Later on images were obtained using Nikon 90i microscope. For quantification of the number of cells immunopositive for a given marker (BrdU or MAP2), in any given experiment, the number of positive cells outside and leaving the NS body were counted using Image J (NIH) and normalized to total nuclei. Cell numbers were estimated from a total of 30 fields (∼7000 cells from 10–15 NS) of three different experiments using a 20x objective.

### Statistical methods

Estimated total number of positive cells from each mouse was used for statistical evaluation of reported differences. Statistical comparisons were made by the Student's *t* test, with *P*≤0.05 being considered significant. All data are presented as mean ± S.E.
